# Beneficial effect of agmatine on brain apoptosis, astrogliosis, and edema after rat transient cerebral ischemia

**DOI:** 10.1186/1471-2210-10-11

**Published:** 2010-09-06

**Authors:** Che-Chuan Wang, Chung-Ching Chio, Ching-Hong Chang, Jinn-Rung Kuo, Ching-Ping Chang

**Affiliations:** 1 Department of Surgery, Chi Mei Medical Center, Tainan 710, Taiwan; 2 Department of Biotechnology, Southern Taiwan University, Tainan 710, Taiwan; 3 Department of Medical Research, Chi Mei Medical Center, Tainan 710, Taiwan

## Abstract

**Background:**

Although agmatine therapy in a mouse model of transient focal cerebral ischemia is highly protective against neurological injury, the mechanisms underlying the protective effects of agmatine are not fully elucidated. This study aimed to investigate the effects of agmatine on brain apoptosis, astrogliosis and edema in the rats with transient cerebral ischemia.

**Methods:**

Following surgical induction of middle cerebral artery occlusion (MCAO) for 90 min, agmatine (100 mg/kg, i.p.) was injected 5 min after beginning of reperfusion and again once daily for the next 3 post-operative days. Four days after reperfusion, both motor and proprioception functions were assessed and then all rats were sacrificed for determination of brain infarct volume (2, 3, 5-triphenyltetrazolium chloride staining), apoptosis (TUNEL staining), edema (both cerebral water content and amounts of aquaporin-4 positive cells), gliosis (glial fibrillary acidic protein [GFAP]-positive cells), and neurotoxicity (inducible nitric oxide synthase [iNOS] expression).

**Results:**

The results showed that agmatine treatment was found to accelerate recovery of motor (from 55 degrees to 62 degrees) and proprioception (from 54% maximal possible effect to 10% maximal possible effect) deficits and to prevent brain infarction (from 370 mm^3 ^to 50 mm^3^), gliosis (from 80 GFAP-positive cells to 30 GFAP-positive cells), edema (cerebral water contents decreased from 82.5% to 79.4%; AQP4 positive cells decreased from 140 to 84 per section), apoptosis (neuronal apoptotic cells decreased from 100 to 20 per section), and neurotoxicity (iNOS expression cells decreased from 64 to 7 per section) during MCAO ischemic injury in rats.

**Conclusions:**

The data suggest that agmatine may improve outcomes of transient cerebral ischemia in rats by reducing brain apoptosis, astrogliosis and edema.

## Background

It is believed that aquaporin-4 (AQP4) is abundantly expressed by astrocytes lining the ependymal and pial surface that are in contact with cerebrospinal fluid in the cerebroventricular system and subarachnoid space [1]. Highly polarized AQP4 expression is also found in astrocytic foot process near or in direct contact with blood vessels [2]. In fact, AQP4, functions as an efficient water-selective transporting protein in the central nervous system [3]. Mice deficient in AQP4 have much better survival than wild-type mice in a model of brain edema caused by acute water intoxication [4]. In another model of brain edema, focal ischemic stroke produced by middle cerebral artery occlusion (MCAO), AQP4-deficient mcie have improved neurological output [4]. These results suggest that AQP4 may play an important role in cerebral ischemia-induced brain edema in the mouse [5]. Inducible nitric oxide synthase (iNOS) has also been shown to participate in the neuronal damage of focal cerebral ischemia [6,7].

Agmatine, formed by the decarboxylation of L-arginine by arginine decarboxylase, is synthesized in the mammalian brain [8]. Several recent evidence have accumulated to indicate that agmatine has neuroprotective effects in many disease models [9-21]. It was shown that agmatine therapy in rodent model of transient focal cerebral ischemia was highly protective against neurological injury [15,21]. A more recent report showed agmatine treatment decreased the AQP4 expression in ischemic mice [22]. However, the mechanisms underlying the neuroprotective effects of agmatine remain unclear.

The aims of the present study was to assess the effects of systemic delivery of agmatine on the MCAO-induced motor and proprioceptive dysfunction 4 days after MCAO to determine whether agmatine therapy would produce beneficial effects on histochemical markers of apoptosis, astrogliosis and edema.

## Methods

### Reagents

Agmatine was used in its sulfate salt form, purchased from Sigma co. Ltd (MO, USA) and dissolved in 0.9% saline for administration.

### Animals and stroke model

Adult male Sprague-Dawley rats (weight, 253 ± 10 g) were housed under controlled environmental conditions with ambient temperature of 22 ± 1°C, relative humidity of 65% and 12-h light/dark cycle, with free access to food and water. Brain focal ischemia was induced by MCAO in rats by intraluminal filament, using the relatively non-invasive technique previously described by Belayev et al [23]. Briefly, rats were anesthetized with sodium pentobarbital (25 mg/kg, i.p.), ketamine (44 mg/kg, i.m.), atropine (0.02633 mg/kg, i.m.), and xylazine (6.77 mg/kg, i.m.). Body temperature was measured with a rectal probe and was kept at 37°C during the surgical procedure with a heating pad. Under an operating microscope, the external and internal right carotid arteries were exposed through a neck incision. The external carotid artery was cut approximately 3 mm above the common carotid artery bifurcation and a silk suture was tied loosely around the external carotid stump. A silicone-coated nylon filament (diameter: 0.37 mm, Doccal Corporation, Redlands, CA, USA) was then inserted into the external carotid artery and gently advanced into the internal carotid artery, approximately 18 mm from the carotid bifurcation, until mild resistance was felt, thereby indicating occlusion of the origin of the middle cerebral artery in the Willis circle. The suture was tightened around the intraluminal filament to prevent bleeding. To allow reperfusion, the nylon filament was withdrawn 90 minutes after MCAO. The animals were allowed to awaken and were kept in their cages with free access to food and water.

### Experimental design

Ninty-six Animals were randomly assigned to sham-operated group (n = 32), MCAO rats treated with saline (1 ml/kg, i.p.) (n = 32), or MCAO rats treated with agmatine (n = 32). All tests were run blinded, and the animal codes were revealed only at the end of the behavioral and histological analyses. Agmatine (Sigma Chemical Co., St. Louis, Mo, USA) was injected (100 mg/kg, i.p.) 5 min after beginning of reperfusion and again once daily for the next 3 post-operative days [21].

All the experimental procedures were carried out in accordance with the National Institutes of Health Guidelines and were approved by the Chi Mei Medical Center Animal Care and Use Committee to minimize the discomfort to the animals during surgery and in the recovery period.

In the rat model, AQP4 mRNA was increased 3 days after ischemia [24]. In the following experiments, all the assessments except behavioral tests were conducted 4 days after MCAO (Table 1).

**Table 1:** Experimental plan

Groups of animals	Maximal angle	%MPE	Infarction volume	Brain water	GFAP positive cells	iNOS expression cells	AQP4 cells	Neuronal apoptosis
1. sham operated rats	n = 8	n = 8	n = 8	n = 8	n = 8	n = 8	n = 8	n = 8
2. (MCAO+saline) rats	n = 8	n = 8	n = 8	n = 8	n = 8	n = 8	n = 8	n = 8
3. (MCAO+Agmatine) rats	n = 8	n = 8	n =8	n =8	n =8	n = 8	n = 8	n = 8

n, numbers of animals used. All measurements were performed 4 days after MCAO or sham operation.

### Motor function test

The inclined plane was used to measure limb strength. Animals were placed, facing right and then left, perpendicular to the slope of a 20 × 20-cm ruffer ribbed surface of an inclined plane starting at an angle of 55° [25,26]. The angle was increased or decreased in 5° increments to determine the maximal angle an animal could hold to the plane. Data for each day were the means of left and right side maximal angle. Measurements of motor deficits were conducted daily but only the 4^th ^day values were presented.

### Cerebral infarction assessment

Rats were with deep anesthesia and were transcardially perfused with heparinized 0.05-mol/L phosphate-buffered saline (PBS) followed by ice-cold 15% sucrose in PBS. The brains were rapidly removed and frozen in liquid nitrogen and then sectioned for immunohistochemistry. Eight serial sections from each brain were cut at 2-mm intervals from the frontal pole using a rat brain matrix (Harvard Apparatus, Holliston, MA, USA). To measure ischemic change, brain slices were stained in a solution containing 2% 2,3,5-triphenyltetrazolium chloride (TTC) in saline, at 37°C. The brain sections stained with TTC were performed in all the three groups. Each group contained 6 animals. After 10 min incubation, the slices were transferred to 5% neutral buffered formaline and stored at 4°C prior to analysis. Infarct volume (mm^3^), as revealed by negative TTC stains indicating dehydrogenase-dificient tissue, was measured in each slice and summed using computerized planimetry (PC-based image tools software). The volume of infarction was calculated as 2 mm (thickness of the slice) × (sum of the infarction area in all brain slices [mm^2^]) [27].

### Brain water contents

Four days after MCAO, rats were killed and brains were removed. The pons and olfactory bulb were removed and the brains were weighted to obtain their wet weight (ww). Thereafter brains were dried at 110°C for 24 h for determing their dry weight (dw). Brain water content was calculated by using the following formula: (ww-dw)/ww × 100 [28] as used as an index for brain edema.

### Immunohistological determination

Adjacent 50-μm sections corresponding to coronal coordinates 0.2 mm to 0.7 mm anterior to the bregma were incubated in 2 mol/L HCL for 30 min, rinsed in 0.1 mol/L boric acid (pH 8.5) for 3 min at room temperature and then incubated with primary antibodies in PBS containing 0.5% normal bovine serum at 4°C overnight. After being washed in PBS, the sections were incubated with secondary antibodies for 1 h at room temperature. The following primary antibodies were used in this study: inducible nitric oxide synthase (iNOS; 1:200 BD bioscience), anti-neuronal-specific nuclear protein (Neu N, 1:200 Roche Diagnostics); GFAP (1:400 Abcam) or AQP4 (1:100 Chemicon) and then detected with Alexa-Fluor^®^, 568 grat anti-mouse (IgG) antibody. The sections were examined using an Olympus B × 51 microscope. The stained cells in ischemic ortex (penumbra) were calculated in 3 coronal sections from each rat and summed and expressed as the mean number per mm^2^. The numbers of labeled cells were counted from the equivalent field for 3 different groups of rats. For negative coronal sections, all procedures were performed in the same manner without the primary antibodies.

### TUNEL assay

Brain tissues were embedded in paraffin and then sectioned for the TUNEL assay. TUNEL assay was used for assessment of apoptosis. The color was developed using 3,3-diamino-benzidine tetrachloride (DAB). The sections were xylene- and ethanol-treated for paraffin removal and for dehydration. They were then washed with PBS and incubated in 3% H_2_O_2 _solution for 20 min. The sections were treated with 5 μg/ml proteinase k for 2 min at room temperature, and re-washed with PBS (0.1 M, pH7.4, PBS). The sections were then treated with a TNLE reaction mixture (nucleotide Rochel Mannheim, Germany) at 37°C for 1 h, and then the sections were washed with distilled water (D/W). They were then re-incubated in an-fluorescein antibody-conjugated with horseradish peroxidase at room temperature for 30 min, re-wash, and then visualized using the avidin-biotin-peroxidase complex (ABC) technique and 0.05% 3,3-diaminobenzidine (DAB, Sigma) as a chromogen. The numbers of TUNEL positive cells were counted by a pathologist in 30 fields/sections (×200 magnification). The blinding was performed for the pathology grading of the results.

### Proprioception

Proprioception evaluation was based on the resting, posture, and postural reactions ("tactile placing" and "hopping") [29]. The functional deficit was graded as 3 (normal or 0% maximal possible effect [MPE], 2 (slightly impaired), 1 (severely impaired), and 0 (completely or 100% MPE) [30]. This test was performed by lifting the front half of the animal off the ground and lifting one hind limb at a time off the ground so that the animal was standing on just one limb. Then, the animal was moved laterally, which normally evoked a prompt hopping response with the weight- bearing limb in the direction of movement to prevent the animal from falling. A predominant motor block would cause a prompt but weaker-than-normal response. Conversely, a predominantly proprioceptive block causes a delayed hopping followed by greater lateral hops to prevent the animal from falling. In the case of full blockade, there would be no hopping maneuvers [31].

### Statistics

One way analysis of variance (ANOVA) test was used for comparing the differences between the groups. When the ANOVA showed significant differences, the post hoc multiple comparison test (Tukey) was applied. The neurological scores were compared by Kruskal-Wallis one way ANOVA followed by multiple comparison procedures by Dunn's method. The data were expressed as means ± standard deviation (S.D.), and P < 0.05 was accepted as statistical significant.

## Results

### Agmatine attenuates MCAO-induced motor deficits

**Figure 1:**
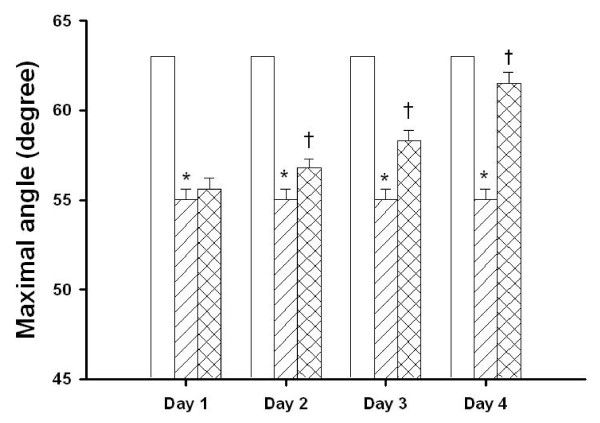
**Daily maximal angle animals could cling to an inclined plane for 8 sham-operated rats (□), 8 vehicle-treated MCAO-injured rats (▨), and 8 agmatine-treated MCAO-injured rats (▩)**. *Maximal grip angle 2-4 days after MCAO was significantly (P < 0.05) decreased for MCAO-injured animals treated with an i.v. dose of normal saline (1 mL/kg) compared with sham controls. ^†^Maximal grip angle 2-4 days after MCAO injury was significantly (P < 0.05) increased for MCAO-injured animals treated with an i.v. dose of agmatine (100 mg/kg) compared with vehicle controls.

Maximal grip angle 1-4 days after MCAO injury was significantly decreased for MCAO-injured animals treated with an i.p. dose of normal saline compared with MCAO sham controls (Fig. 1). The maximal grip angle 2-4 days after MCAO injury was significantly increased for MCAO-injured animals treated with an i.p. dose of agmatine compared with vehicle controls (62 degrees vs 54 degrees; Fig. 1).

### Agmatine attenuates MCAO-induced proprioception blockade

**Figure 2:**
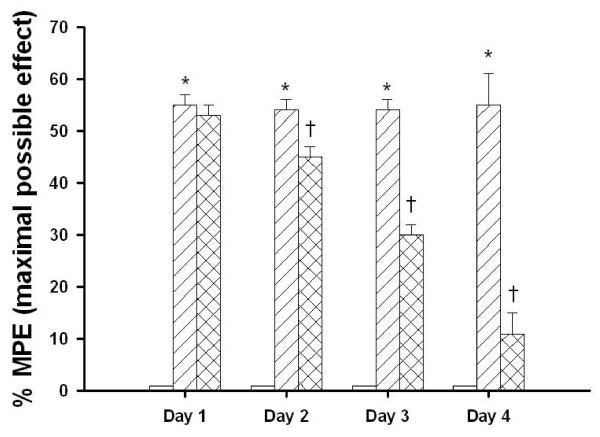
**Daily percentage of maximal possible effect (%MPE) of proprioception blockade by 8 sham-operated rats (□), 8 vehicle-treated MCAO-injured rats (▨), and 8 agmatine-treated MCAO-injured rats (▩)**.*The percent of MPE 2-4 days after MCAO injury was significantly (P < 0.05) increased for MCAO-injured animals treated with an i.v. dose of normal saline (1 mL/kg) compared with MCAO sham controls. ^†^The percentage of MPE 2-4 days after MCAO injury was significantly decreased for MCAO-injured animals treated with an i.v. dose of agmatine (100 mg/kg) compared with vehicle controls.

The percentage of MPE or proprioception blockade 1-4 days after MCAO injury was significantly increased for MCAO-injured animals treated with an i.p. dose of normal saline compared with MCAO sham controls (Fig. 2). The MCAO-induced proprioception blockade 2-4 days after MCAO injury was significantly reversed by an i.p. dose of agmatine (100 mg/kg; p < 0.05) (10% MPE vs 54% MPE; Fig. 2).

### Agmatine attenuates MCAO-induced cerebral infarction volume

**Figure 3:**
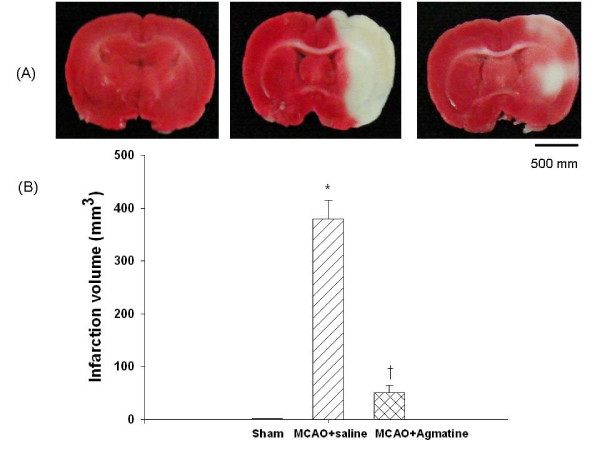
**Infarction volume (B) by MCAO in rats (n = 8 in each group of different treatments)**. *The infarction volume 4 days after MCAO injury was significantly (P < 0.05) increased for MCAO-injured animals treated with an i.v. dose of normal saline (1 mL/kg) compared with MCAO sham controls. ^†^The infarction volume 4 days after MCAO-injury was significantly decreased for MCAO-injured animals treated with an i.v. dose of agmatine (100 mg/kg) compared with vehicle controls. The photomicrograph (A) illustrates the infarction for a sham-operated rat, a MCAO rat treated with normal saline, and a MCAO rat treated with agmatine. Triphenyltetrazolium chloride staining shows severe infarctions characterized by pale TTC stains (whitish color) in the cortex and striatum on all brain sections examined.

The cerebral infarction volume 4 days after MCAO injury was significantly increased for MCAO-injured animals treated with an i.p. dose of normal saline compared with MCAO sham controls (Fig. 3). The infarction volume 4 days after MCAO injury was significantly reduced for MCAO-injured animals treated with an i.p. dose of agmatine compared with vehicle controls (370 mm^3 ^vs 50 mm^3^) (Fig. 3).

### Agmatine attenuates MCAO-induced cerebral edema

**Figure 4:**
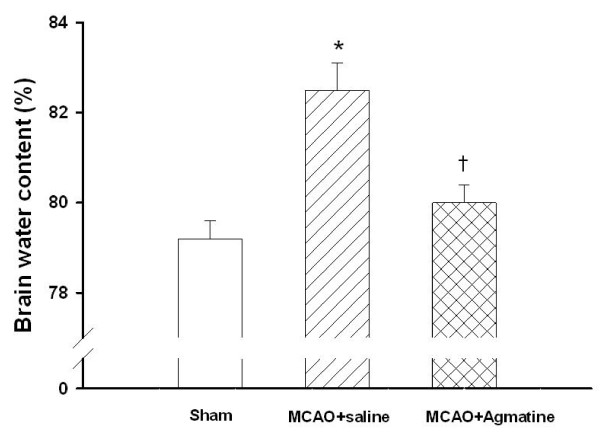
**Cerebral edema by MCAO in rats (n = 8 in each group of different treatments)**. *The brain water content 4 days after MCAO injury was significantly (P < 0.05) increased for MCAO-injured animals treated with an i.v. dose of normal saline (1 mL/kg) compared with MCAO sham controls. ^†^The cerebral water content 4 days after MCAO injury was significantly decreased for MCAO-injured animals treated with an i.v. dose of agmatine (100 mg/kg) compared with vehicle controls.

The brain water content 4 days after MCAO injury was significantly increased for MCAO-injured animals treated with an i.p. dose of normal saline compared with MCAO sham controls (Fig. 4). The cerebral water content 4 days after MCAO injury was significantly decreased for MCAO-injured animals treated with an i.p. dose of agmatine compared with vehicle controls (82.5% vs 79.4%; Fig. 4).

### Agmatine attenuates MCAO-induced cerebral gliosis

**Figure 5:**
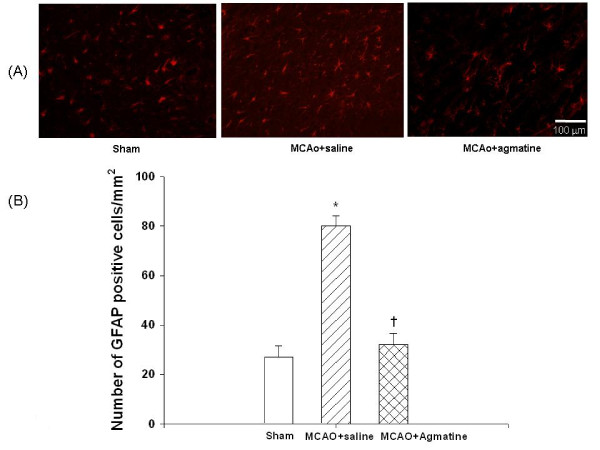
**Gliosis by MCAO in rats (n = 8 in each group of different treatments)**. *The number of GFAP-positive cells (B) 4 days after MCAO injury was significantly (P < 0.05) increased for MCAO-injured animals treated with an i.v. dose of normal saline (1 ml/kg) compared with MCAO sham controls. ^†^The increased number of GFAP-positive cells 4 days after MCAO injury was significantly decreased for MCAO-injured animals treated with an i.v. dose of agmatine (100 mg/kg) compared with vehicle controls. The photomicrograph (A) illustrates a typical example for a sham-operated control, a vehicle-treated MCAO rat, and an agmatine-treated MCAO rat.

The numbers of GFAP-positive cells 4 days after MCAO injury was significantly increased for MCAO-injured animals treated with an i.p. dose of normal saline compared with MCAO sham controls (Fig. 5). The increased numbers of GFAP-positive cells 4 days after MCAO injury was significantly reversed for MCAO-injured animals treated with an i.p. dose of agmatine compared with vehicle controls (100 GFAP-positive cells vs 20 GFAP-positive cells per section; Fig. 5).

### Agmatine attenuates MCAO-induced overexpression of iNOS

**Figure 6:**
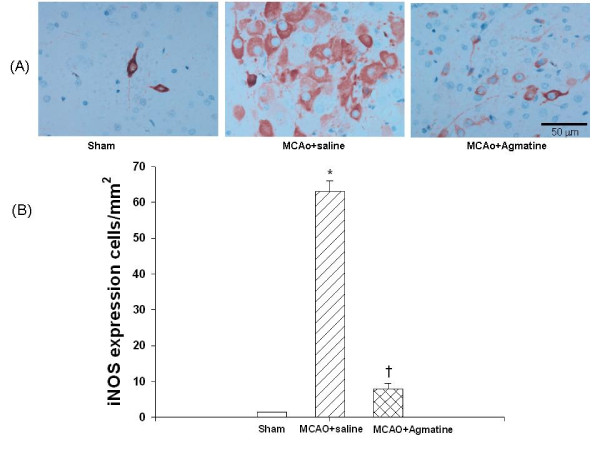
**Overexpression of cerebral iNOS by MCAO in rats (n = 8 in each group of different treatments)**. *The numbers of iNOS-positive cells (B) 4 days after MCAO injury was significantly (P < 0.05) increased for MCAO-injured animals treated with an i.v. dose of normal saline (1 mL/kg) compared with MCAO sham controls. ^†^The increased numbers of iNOS-positive cells 4 days after MCAO-injury was significantly decreased for MCAO-injured animals treated with an i.v. dose of agmatine (100 mg/kg) compared with vehicle controls. The photomicrograph (A) illustrates a typical example for a sham-operated control, a vehicle-treated MCAO rat, and an agmatine-treated MCAO rat.

The numbers of iNOS-positive cells 4 days after MCAO injury was significantly increased for MCAO-injured animals treated with an i.p. dose of normal saline compared with MCAO sham controls (Fig. 6). The increased numbers of iNOS-positive cells 4 days after MCAO injury was significantly decreased for MCAO-injured animals treated with an i.p. dose of agmatine compared with vehicle controls (64 iNOS-positive cells vs 7 iNOS-positive cells per section; Fig. 6).

### Agmatine attenuates MCAO-induced neuronal apoptosis

**Figure 7:**
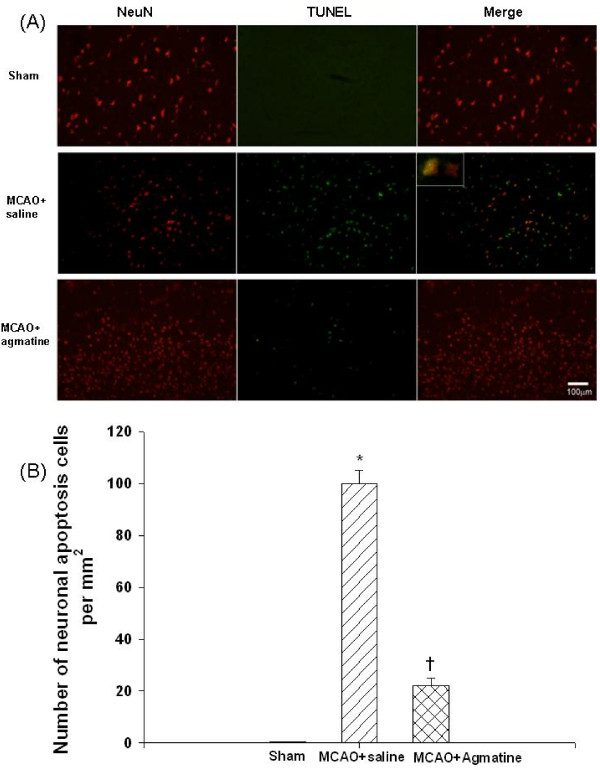
**Neuronal apoptosis by MCAO in rats (n = 8 in each group of different treatments)**. *The numbers of neuronal apoptosis cells (B) 4 days after MCAO injury was significantly (P < 0.05) increased for MCAO-injured animals treated with an i.v. dose of normal saline (1 mL/kg) compared with MCAO sham control. ^†^The increased numbers of neuronal apoptosis cells 4 days after MCAO injury was significantly decreased for MCAO-injured animals treated with an i.v. dose of agmatine (100 mg/kg) compared with vehicle controls. The photomicrographs (A) illustrates a typical example for a sham-operated control, a vehicle-treated MCAO rat, and an agmatine-treated MCAO rat. A cell with NeuN-TUNEL double positive was recognized as a neuronal apoptosis cell.

The numbers of neuronal apoptosis cells 4 days after MCAO injury was significantly decreased for MCAO-injured animals treated with an i.p. dose of normal saline compared with MCAO sham controls (Fig. 7). The decreased numbers of neuronal apoptosis cells 4 days after MCAO injury was significantly decreased for MCAO-injured animals treated with an i.p. dose of agmatine compared with vehicle controls (100 vs 20 cells per section; Fig. 7).

### Agmatine attenuates MCAO-induced overexpression of AQP4

**Figure 8:**
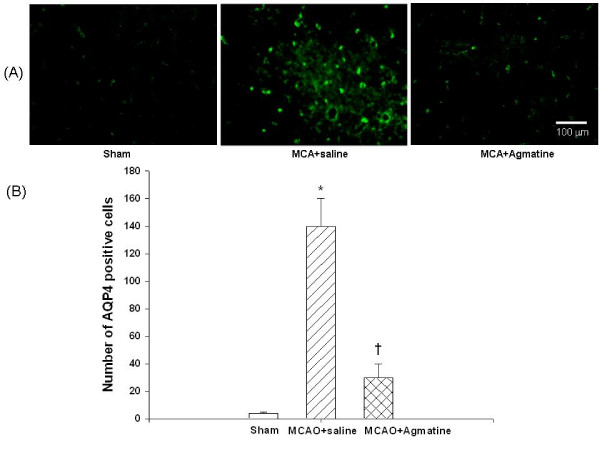
**Overexpression of cerebral AQP4 by MCAO in rats (n = 8 in each group of different treatments)**. *The numbers of AQP4-positive cells (B) 4 days after MCAO injury was significantly (P < 0.05) increased for MCAO-injured animals treated with an i.v. dose of normal saline (1 mL/kg) compared with MCAO sham controls. ^†^The increased numbers of AQP4-positive cells 4 days after MCAO injury was significantly decreased for MCAO-injured animals treated with an i.v. dose of agmatine (100 mg/kg) compared with vehicle controls. The photomicrograph (A) illustrates a typical example for the beneficial effect of agmatine.

The numbers of cerebral AQP4-positive cells 4 days after MCAO injury was significantly increased for MCAO-injured animals treated with an i.p. dose of normal saline compared with MCAO sham controls (Fig. 8). The increased numbers of cerebral AQP4-positive cells 4 days after MCAO injury was significantly decreased for MCAO-injured animals treated with an i.p. dose of agmatine compared with vehicle controls (140 vs 84 AQP4-positive cells per section; Fig. 8).

## Discussion

Agmatine's effect on reducing the edema formation after cerebral ischemia was already been reported but the results suggesting the agmatine's therapeutic potential was convincing [15,21,22]. Our results demonstrate that agmatine therapy reduces neuronal apoptosis, gliosis, and edema in an animal model of stroke followed by reperfusion. In particular the MCAO-induced overexpression of cerebral AQP4 can be greatly reduced by agmatine therapy, suggesting that the enhanced water movement associated with the pathophysiology of cerebral edema after transient cerebral ischemia is attenuated by agmatine. It is believed that AQP4 is abundantly expressed by astrocytes lining the ependymal and pial surface that are in contact with cerebrospinal fluid in the cerebroventricular system and subarachnoid space [1]. Highly polarized AQP4 expression is also found in astrocytic foot process near or in direct contact with blood vessels [2]. As demonstrated in the current results, the MCAO-induced overexpression of cerebral AQP4 can be attenuated following systemic delivery of agmatine in rats. Moreover agmatine treatment decreased the AQP4 expression in ischemic mice [22]. Evidence indicates that AQP4 functions as an efficient water-selective transporting protein in the central nervous system [3]. Mice deficient in AQP4 have much better survival than wild-type mice in a model of brain edema caused by acute water intoxication [4]. In another model of brain edema, focal ischemic stroke produced by MACO, AQP4-deficient mice have improved neurological output [4].

In the present study, histoimmunological evaluation reveals that, at 4 days after MCAO, gliosis (evidenced by increased numbers of both GFAP-positive and AQP4-positive cells) and neuronal apoptosis (evidenced by increased numbers of NeuN and TUNEL double positive cells) in the ischemic cortex, can be greatly reduced by agmatine therapy. The reduction of both gliosis and neuronal apoptosis in agmatine-treated ischemic animal is paralled by the reduced infarct volume and near normal motor and proprioceptive function. Thus, it appears that agmatine may protect against the delayed infarct expansion caused by activated astrocytes during transient cerebral ischemia. Astrocytes are believed to be responsible for most glutamate uptake in synaptic areas and consequently are the major regulators of glutamate homeostasis [32]. Microglia in turn may secrete cytokines, which can impair glutamate uptake. Finally, oligodendrocytes, the myelinating cells of the central nervous system, are very sensitive to excessive glutamate signaling, which can lead to the apoptosis or necrosis of these cells [32]. Agmatine, an inhibitor of N-methyl-D-aspartate (NMDA) glutamate receptors [33], may also protect against cerebral ischemia injury by reducing glutamate-mediated glial injury. In addition, it has been shown that agmatine can rescue astrocytes from death caused by ischemic and/or ischemia-perfusion neuronal injury in vitro [34].

Agmatine is stored and synthesized in astrocytes [35]. Arginine decarboxylase, the enzyme responsible for synthesis of agmatine, is largely localized in astrocytes, so endogenous agmatine production is likely in astrocytes. Abe et al [36] have reported that agmatine works to protect neurons by inhibiting the production of nitric oxide in microglia. During the initial phase of ischemia, enhanced nitric oxide generated by endothelial nitric oxide synthase (eNOS) maintains cerebral blood flow, while nitric oxide generated from neuronal nitric oxide synthase (nNOS) is neurotoxic [37,38]. Like nNOS, the induction of inducible nitric oxide synthase (iNOS) can participate in neuronal damage occurring in the late phase of focal cerebral ischemia [6,7]. Prior studies have shown that agmatine reduces the production of nitric oxide and the expression of nNOS but not iNOS in vitro. However, in vivo, agmatine inhibites the expression of both nNOS and iNOS in a mouse model of transient focal cerebra ischemia [29]. In vitro study has also demonstrated that agmatine inhibits the production of nitric oxide by decreasing the activity of iNOS in astroglial cells [39]. In the current study, in a rat model of transient cerebral ischemia, we further show that agmatine inhibits the overexpression of cerebral iNOS after MCAO. These observations prompted us to think that overproduction of NO from eNOS protects brain tissue by maintaining regional cerebral blood flow; however, NO production from either nNOs or iNOS leads to neurotoxicity. Thereafter, agmatine may reduce cerebral ischemia injury by inhibiting the detrimental effects of both iNOS and nNOS.

Similar kind of results were published previously stating agmatine treatment lowered the expression of iNOS in the early post ischemic MCAO mice and agmatine had no effect at the later period of reperfusion [15]. These findings are not supported by both previous [2,39] and present results showing a significant less expressing of iNOS in agmatine treatment group at the later period of reperfusion. The reason for the discrepancy between the two groups of data are not apparent now.

Finally, it should be mentioned that AQP4 phosphorylation is related to nitric oxide in astrocyte [40]. It is possible that agmatine would reduce the AQP4 expression in astrocyte by inhibiting nitric oxide synthase [41,42].

## Conclusions

In summary, our current findings revealed that agmatine therapy reduced cerebral infarct volume by 87%, brain edema formation by 50% and functional deficits by 70% 4 days after transient cerebral ischemia in the rat. In addition, both cerebral gliosis (evidenced by overexpression of both GFAP and AQP4) and neuronal apoptosis (evidenced by increased numbers of NeuN plus TUNEL double positive cells) and neurotoxicity (evidenced by increased iNOS expression) that occurred during transient cerebral ischemia could be greatly attenuated by agmatine therapy. It is likely that agmatine therapy improved the neurological outcome after transient cerebral ischemia by reducing neuronal apoptosis, gliosis, neurotoxicity and cerebral edema formation in the rat.

## Key messages

• An intraperitoneal injection of agmatine attenuates MCAO-induced motor deficits and proprioception blockade.

• Agmatine therapy attenuates MCAO-induced cerebral infarction, edema, gliosis, and neuronal apoptosis.

• Agmatine therapy attenuates MCAO-induced overexpression of brain iNOS and AQP4.

## Abbreviations

MCAO: middle cerebral artery occlusion; GFAP: glial fibrillary acidic protein; AQP4: aquaporin-4; TTC: triphenyltetrazolium chloride; TUNEL: deoxynucleotidyl-transferase-mediated and duDP-biotin Nick end- labeling; INOS: inducible nitric oxide synthase; NEUN: neuronal-specific-nuclear protein.

## Competing interests

The authors declare that they have no competing interests.

## Authors' contributions

CCW and CCC carried out the study on rat transient cerebral artery occlusion and drafted the manuscript. CCW, CHC and CPC carried out the histobiochemical assay CCC, MTL, and CPC conceived the study and participated in its design and coordination and helped drafting the manuscript. All authors read and approved the final manuscript.
